# Biochemical Applications of Chromatography/SIMS

**DOI:** 10.6028/jres.093.134

**Published:** 1988-06-01

**Authors:** K. L. Busch, M. S. Stanley, K. L. Duffin, J. C. Dunphy

**Affiliations:** Department of Chemistry, Indiana University, Bloomington, IN 47405

The balanced capabilities of a high resolution separation method coupled with a flexible detection method such as mass spectrometry results in powerful analytical methods including gas chromatography/mass spectrometry and liquid chromatography/mass spectrometry. However, separations for diverse applications such as organic synthesis, pharmacological analysis, and biochemical separations are still based on static chromatography, defined as a method in which the separation of sample components is based on a spatial separation, rather than on a difference in retention times. Although capabilities for separation of very complex mixtures for static chromatography (thin layer or paper chromatography or electrophoresis) do not rival the very high resolution achievable with the gas and liquid chromatography, the resolution is adequate for a large number of applications, and the ease and low relative cost of the methods are advantageous. General detection methods for organic compounds separated by these forms of chromatography still seem relatively crude. Color development, charring, staining, or fluorescence measurements provide the location of sample spots, but little information about sample identity. In many cases, sample identity is suggested on the basis of an *R*_f_ identical with that of the standard. Such a match is a necessary but not a sufficient means of compound identification.

Over the past 2 years, we have constructed a secondary ion mass spectrometer (SIMS) that accommodates large chromatograms within the source, and sputters organic ions from the surfaces to produce the mass spectrum of samples directly from the chromatogram. Details of instrument construction have been reported [1]. In essence, the chromatogram is moved in the *x* and *y* axes into and out of the point of instrument focus, defined as the locus of the primary ion beam, and the secondary ion extraction optics of a quadrupole mass spectrometer. Data in four dimensions are generated: *x, y, m/z* ratio, and relative abundances of the ions in the mass spectrum [2–4].

Analytical advantages of the chromatography/SIMS instrument include an independent access order to sample spots, variable integration time for spectral measurement, variable spatial resolution adjusted in real time to maximize analysis efficiency, the ability to store sample as well as data generated in its analysis, preservation of all sample applied to the chromatogram, the ability to analyze chromatograms from outside sources, and the ability to calibrate the instrument with each chromatogram [5]. We have demonstrated application of chromatography/SIMS to a variety of biochemical samples and have used the mass spectral data to identify compounds contained in the chromatogram, and to increase the resolution of the chromatographic separation. The mass spectrometer is a “biosensor” that can either be operated as a general detector, or in concert with functional-group-specific-derivatization reactions that have been concurrently developed.

Figure 1 illustrates part of the data array measured in an analysis of a mixture of chenodeoxycholic add (A) and cholic add (B) separated by thin layer chromatography (metal-backed silica gel plate, 20 pm thickness). A dominant ion in the positive ion SIMS of chenodeoxycholic acid appears at *m/z* 357; similarly, the SIMS spectrum of cholic add contains an abundant ion at *m/z* 355. The mass spectral data acquired over the range of *x* and *y* coordinates is reconstructed for ions of these masses. These data were acquired with a cesium ion gun with moderate spatial resolution. The maximum sample spot diameter is 1 mm, and 5 μg of each compound were present in the spot. Spectra could be recorded for several hours in each analysis (sample consumption rate is a few tens of picograms per second), and the same distribution is recorded if the chromatogram is removed from the mass spectrometer, stored, and reanalyzed later.

Figure 2 represents the analysis of pyridostigmine bromide separated by thin layer chromatography. The structure of this drug is shown; the SIMS spectrum contains an abundant ion for the intact cation at *m/z* 223. Variation of this particular ion with *x* and *y* is shown in the abundance plot. The same data are shown plotted as isoabundance contours. Each ion in the SIMS spectrum derived from the sample exhibits similar contours, and pattern recognition programs can be used to link such ions together to form a mass spectrum of the compound free of background.

The chromatography/SIMS method has been extended to the indirect analysis of electrophoretograms. Although aqueous gels can be dried for analysis, this often degrades the spatial resolution of the separation and makes the gel difficult to handle. The high vapor pressure of water (even at low temperatures) precludes direct analysis of electrophoretic gels by SIMS. Standard sample transfer techniques such as Southern and Western blots are used to transfer samples to nitrocellulose and diazotized media, then analyzed by SIMS. Bradykinin and a series of related peptides have been so separated and analyzed; the SIMS spectra obtained are identical to those obtained from a discrete sample.

Control of the physical and chemical nature of the chromatographic surface underlies the success of chromatography/SIMS. For maximum sensitivity, the sample should be in an ionic state, and should exhibit surfactant properties in the phase transition matrix used for the analysis [4]. Figure 3 is the positive ion SIMS spectrum of a mixture of five pyrylium salts (10 μg each) used for derivatization of primary amine groups in peptides. The recorded spectrum contains only ions from the pyrylium salt of the structure shown, which was synthesized specifically for Us surface activity. No ions from the other pyrylium salts, or from the matrix, are observed. Several peptides have been derivatized with this surface-active agent. In addition to increased sensitivity, determination of the sequence of the peptide by MS/MS analysis of the parent ion of the derivative is greatly simplified.

## Figures and Tables

**Figure 1:**
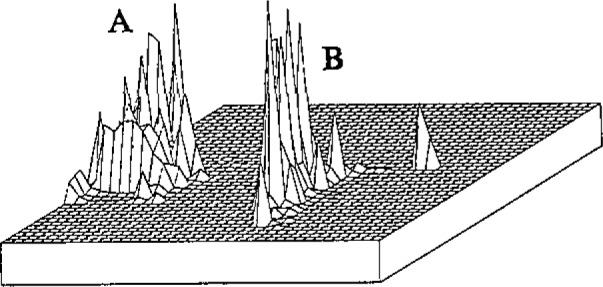
Reconstruction of the abundances of the ions at *m/z* 357 (chenodeoxycholic acid, A) and *m/z* 355 (cholic acid, B) as a function of *x* and *y* coordinates of the thin layer chromatogram on which they were separated.

**Figure 2:**
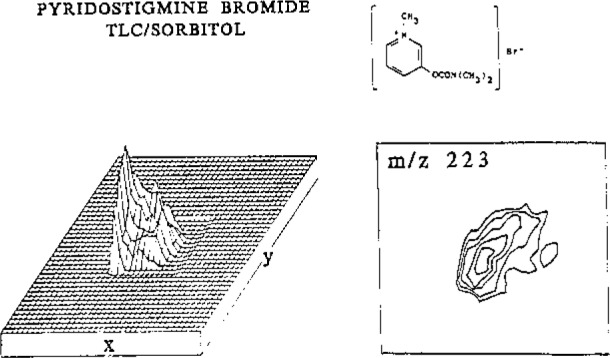
Abundance and isoabundance contour plots of the ion at *m/z* 223 corresponding to the intact cation of pyridostigmine bromide, separated from related drugs in a silica gel thin layer chromatogram.

**Figure 3:**
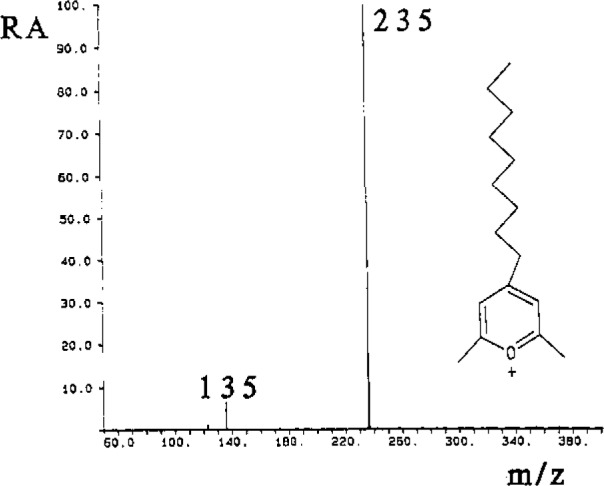
Positive ion secondary ion mass spectrum of a mixture of five pyrylium salts of equal concentration, showing only the ions corresponding to the pyrylium salt of the structure shown, illustrating the surfactant properties of this salt in the matrix used.
